# Surface texture and wear characteristics of micro-hybrid and nano-hybrid resin composites under simulated frictional conditions

**DOI:** 10.1038/s41598-026-49158-y

**Published:** 2026-04-19

**Authors:** Magdalena A. Osiewicz, Cornelis J. Kleverlaan, Arie Werner, Michael Marxer, Dominique Filipec, Magdalena Niemczewska-Wójcik

**Affiliations:** 1 Department of Integrated Dentistry, Jagiellonian University, Cracow, Poland; 2 Department of Dental Materials Science, University of Amsterdam and Vrije Universiteit Amsterdam, Academic Centre for Dentistry Amsterdam (ACTA), Amsterdam, Netherlands; 3 Institute for Microtechnology and Photonics, Eastern Switzerland University of Applied Sciences, Buchs, Switzerland; 4 Faculty of Mechanical Engineering, Cracow University of Technology, Cracow, Poland

**Keywords:** resin-based composites, surface topography, surface morphology, two-body wear, three-body wear, wear rate, Engineering, Materials science

## Abstract

The goal of work was to evaluate the surface texture and wear behaviour of two resin-based composites – AP-X (micro-hybrid) and FSE (nano-hybrid), under two frictional conditions. Specimens were subjected to simulated occlusal wear using the ACTA wear machine, following two-body wear (full contact) and three-body wear (slurry-lubricated) protocols. Surface texture was assessed before and after wear using 3D profilometry and SEM. Roughness parameters and wear rate were measured. SEM micrographs provided insights into wear mechanisms. Initial surface roughness of FSE was higher than AP-X due to nanocluster morphology (Ra: 0.965–1.050 μm vs. 0.470–0.489 μm; Sq: 1.25–1.32 μm vs. 0.554–0.667 μm ). Two-body wear caused significant degradation in FSE, including nanocluster plucking and matrix cracking, whereas AP-X showed more stable wear resistance. Under three-body wear, FSE demonstrated a polishing effect (Ra reduced to 0.564 μm; Sq reduced to 0.766 μm), while AP-X experienced increased surface roughness (Ra increased to 0.966 μm; Sq increased to 0.869 μm) due to macro-filler exposure. Wear analysis confirmed higher material loss under three-body friction conditions. FSE exhibited better performance under slurry-lubricated conditions but was more susceptible to fatigue-driven degradation in full contact. Conversely, AP-X demonstrated superior stability under two-body wear, making it more suitable for bruxers.

## Introduction

Resin-based composites (RBCs) are cornerstone materials in modern restorative dentistry, valued for their aesthetic qualities, adhesive properties, and continuously improving mechanical performance^1^. Among the available formulations, micro-hybrid, and nano-hybrid composites remain two of the most widely used, primarily distinguished by their filler architecture and resin matrix composition.

Micro-hybrid composites typically contain a mixture of micro-sized and fine inorganic fillers (approximately 0.2–3.0 μm) dispersed within a high-viscosity resin matrix, which are commonly based on bisphenol A-glycidyl methacrylate (Bis-GMA) and modified with diluent monomers such as triethylene glycol dimethacrylate (TEGDMA) or urethane dimethacrylate (UDMA). Those composites are characterized by a high filler loading (approximately 85–87 wt%), which contributes to enhanced strength and radiopacity, featuring particularly advantageous in posterior restorations subjected to high occlusal loads^2^. However, the relatively larger filler particles may compromise long-term polish retention and increase surface roughness over time^3^.

In contrast, nano-hybrid composites incorporate nanoscale fillers (typically 20–50 nm) in combination with conventional filler particles, forming a hybrid filler network designed to balance mechanical durability with superior surface smoothness. These materials also employ a Bis-GMA–based resin matrix, frequently modified with UDMA and TEGDMA to improve handling characteristics^4^. The presence of nanosized fillers enhances polish retention and resistance to surface degradation while maintaining adequate mechanical strength^5^.

Although both composite classes demonstrate favourable clinical performance, their long-term resistance to wear and surface degradation, particularly under high-stress, or parafunctional conditions such as bruxism, remains a critical determinant of clinical success. In such situations, factors including filler–matrix interfacial bonding, topographical stability, and resistance to micro-abrasion directly influence restoration longevity^1^.

The durability of a dental tribological system not only depends on material composition, but also on mechanical interactions occurring at contacting surfaces^6,7^, just as in technical systems^8^. Therefore, the accurate characterization of wear behaviour requires a dual methodological approach^9^: (1) metrological analysis of surface topography, both before and after wear, to quantify surface roughness and structural degradation; and (2) tribological testing under simulated oral conditions, employing both two-body (full contact) and three-body (abrasive- or tribofilm) wear protocols^10^.

Despite the clinical relevance of these parameters, relatively few studies systematically integrate advanced surface metrology with controlled tribological simulations. This gap limits current understanding of how wear mechanisms^11^ develop under different types of occlusal contact.

Therefore, the aim of this in vitro study is to evaluate and compare the surface texture characteristics and wear behaviour of two representative resin-based composite systems: a micro-hybrid composite with a high filler load and larger filler particles (Clearfil AP-X) and a nano-hybrid composite containing nanocluster fillers (Filtek Supreme XTE). By combining surface topography analyses (non-contact optical 3D measuring system), surface morphology (scanning electron microscopy SEM), and simulated wear testing, this study seeks to clarify how filler structure and frictional conditions influence degradation patterns, thereby supporting more informed material selection for high stress-bearing restorations. The comprehensive methodological approach for characteristic of resin composites presented in this work has not previously been reported in detail and therefore it could represent a novel contribution.

## Materials and methods

The materials investigated were two commercial resin-based dental composites (RBCs), Clearfil AP-X (AP-X) and Filtek Supreme XTE (FSE), intended for use in load-bearing restorations subjected to masticatory forces. The tested composites differed in filler type, particle size, and filler loading. The micro-hybrid composite AP-X contained a combination of barium glass, silica, and colloidal silica fillers, with an average particle size of approximately 3 μm and a high filler loading of 86 wt%. In contrast, the nano-hybrid composite FSE incorporated zirconia and silica fillers with significantly smaller particle sizes ranging from 0.011 to 0.020 μm and a lower overall filler content of 78 wt%. The materials used in the tests, together with their batch numbers, are presented in Table 1.

**Table 1:** Properties of the materials used in the experiment according to the manufacturer`s data.

Code	Material	Matrix/ Fillers	Type	Filler [wt%]	Size [µm]	Batch/ exp/colour
**AP-X**	Clearfil AP-X	Bis-GMA, TEGDMA, silanated barium glass filler, silanated silica filler, silanated colloidal silica, dl- Camphorquinone	Micro-hybrid Composite	86	0.2-3	0557AA 2016-05/A2
**FSE**	Filtek Supreme XTE	Bis-GMA, UDMA, TEGDMA, bis-EMA, zirconia filler, silica filler	Nano-hybrid composite	78	0.011–0.020	NY33738 2015-08/A2

The resin composites (AP-X and FSE) were stored under standardized conditions (constant room temperature, protected from light) in their original, sealed packaging until the start of the test. Prior to specimen preparation, the materials were inspected for structural integrity and handling properties, and all curing procedures were verified to ensure optimal polymerization, consistent with the manufacturers’ specifications.

Specimens fabricated/machined from both composites were evaluated in terms of surface morphology (qualitative analysis: visualization of surface features) and surface topography (qualitative analysis: axonometric 3D view and profile 2D view; quantitative analysis: surface texture parameters).

**Fig. 1:**
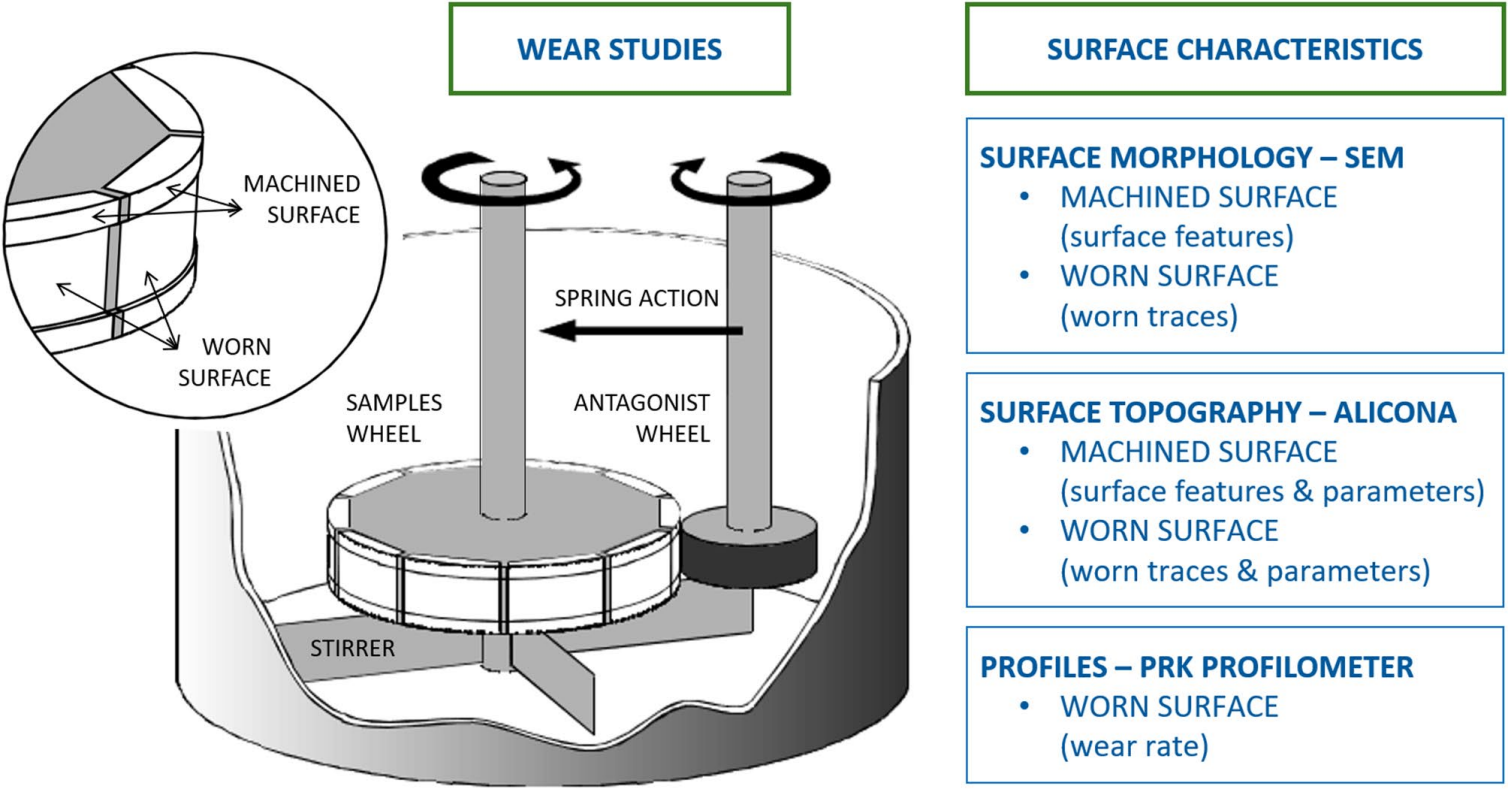
Research methodology.

The tribological performance was assessed using the ACTA wear machine developed at the Academic Centre for Dentistry Amsterdam^12^. Two types of wear were simulated: two-body wear (full contact) and three-body wear (lubricated contact with an abrasive medium)^13^. After the wear tests, the worn surfaces were characterized by scanning electron microscopy (SEM) to evaluate surface morphology and wear features, as well as by non-contact optical 3D measuring system to evaluate surface topography (axonometric/profile view and surface texture parameters). Quantitative mean wear rate (WR) was also determined. A schematic overview of the experimental design is presented in Fig. 1.

The ACTA wear machine effectively replicates the complex mechanisms of the oral cavity. Two distinct protocols were employed to ensure a comprehensive evaluation: the three-body wear protocol, simulating the abrasive and erosive action of food particles (slurry) during mastication, and the two-body wear protocol, simulating direct tooth-to-tooth contact (sliding wear/fatigue) in a liquid environment. The validity of this experimental setup was supported by its high clinical correlation (*r* = 0.90) with long-term in vivo data, as established in prior validation studies^12^. To ensure reproducibility, the specific experimental conditions (wear test parameters) were standardized as shown in Table 2.

**Table 2:** Test conditions for the wear simulation.

Parameter	Specification / Condition
Contact Load	15 N (constant spring force)
Frequency	1.0 Hz (simulated chewing frequency)
Total Cycles	200,000 cycles
Slip Ratio	15% (difference in circumferential speed)
Three-body Medium	slurry of rice and millet seed shells in buffer (pH 7.0)
Two-body Medium	demineralized water
Antagonist Wheel	extra-hardened stainless steel (Ø 19 mm)
Specimen Wheel	composite specimens mounted in a circular array (Ø 48 mm)

The ACTA wear machine is equipped with two wheels of different diameters: the specimen wheel (48 mm diameter) and the stainless-steel antagonist wheel (19 mm diameter). Both wheels rotate in the same direction, with a circumferential speed difference of 15%. The detailed description of the device and testing protocol has been reported elsewhere^12,14^. The two-body wear was generated by direct contact between the specimen wheel and the antagonist wheel in demineralized water. The three-body wear was produced by introducing an abrasive slurry as a third body between the wheels. The slurry consisted of rice and millet seed shells suspended in water (pH = 7) and was contained within a bowl surrounding both wheels. The specimen wheel accommodated inserts of both tested composites: Clearfil AP-X and Filtek Supreme XTE; the remaining positions were filled with a dummy material. The stainless-steel antagonist wheel had an extra-hardened outer surface, in accordance with the standard ACTA wear protocol^12^ and ISO/TS 14,569. Prior to testing, both the specimen wheels and the antagonist wheels were stored in water for two months. During the entire experimental period, the specimen wheels were kept immersed in water at room temperature. The diameter of the antagonist wheel was measured at the beginning and at the end of the two-body wear test. During testing, the antagonist wheel was pressed against the specimen wheel with a spring force of 15 N. Each experimental run consisted of 200,000 cycles (approximately 55.5 h) at a rotational speed of 1 Hz. After each run, ten profilometric measurements were performed at fixed positions on the worn surfaces, perpendicular to the direction of motion, using a contact profilometer (PRK profilometer No. 20702, Perthen GmbH, Hannover, Germany) to determine material loss. Mean wear rate WR values and standard deviations were calculated from these profiles. In accordance with the ACTA protocol established by De Gee and Pallav^12^, the wear of the stainless steel antagonist is considered negligible under the applied load and cycle count. Preliminary and post-test measurements confirmed that there was no significant change in the antagonist’s diameter and surface texture parameters, ensuring consistent frictional conditions throughout the testing of all specimens. The antagonist wear was not studied because it did not impact the relative wear rates of the tested composites.

The machined and worn surface morphologies of AP-X and FSE specimens were examined using a scanning electron microscope (SEM) at ×2,000/50,000 magnification and an accelerating voltage of 10 kV (EVO LS15, Carl Zeiss, Oberkochen, Germany). Prior to the SEM observation, all specimens were gold-sputtered to ensure electrical conductivity.

Surface topography characterization of both machined and worn specimens was performed using a non-contact optical 3D measuring system based on focus-variation technology (Alicona G5, Bruker, Germany) with the following parameters: vertical resolution 50 nm and objective lens 20×/0.40 B EPI. Each specimen was measured in at least three different surface locations. Based multiple measurements the surface texture parameters were obtained, the mean values and standard deviation.

Due to the repeatability of the obtained results of surface texture studies, the representative data are presented, including surface morphology, surface topography 3D views, profile 2D views, and two-dimensional and three-dimensional surface texture parameters, showing the main differences between tested materials/surfaces (machined and worn).

## Results

### Machined surface texture

The surface texture analysis of the machined specimens (Tables 3 and 4) revealed distinct differences between the two investigated materials. Qualitative 2D view (profile) and 3D view (surface topography) representations as well as quantitative surface texture parameters demonstrated that FSE exhibited markedly higher roughness than AP-X.

**Table 3:** Machined surfaces texture characteristics (specimens cooperated in two-body wear protocol).

AP-X						FSE					
3D view (surface topography)											
	
**2D view (profile)**											
	
**Surface texture parameters**											
Ra [µm]	Sq [µm]	Sp [µm]	Sv [µm]	Ssk [-]	Sku [-]	Ra [µm]	Sq [µm]	Sp [µm]	Sv [µm]	Ssk [-]	Sku [-]
0.489 ± 0.056	0.667 ± 0.013	1.93 ± 0.03	1.84 ± 0.03	0.266 ± 0.070	2.63 ± 0.14	0.965 ± 0.053	1.25 ± 0.09	2.46 ± 0.04	2.89 ± 0.05	-0.173 ± 0.197	1.80 ± 0.22

**Table 4:** Machined surfaces texture characteristics (specimens cooperated in three-body wear protocol).

AP-X						FSE					
**3D view (surface topography)**											
	
**2D view (profile)**											
	
**Surface texture parameters**											
Ra [µm]	Sq [µm]	Sp [µm]	Sv [µm]	Ssk [-]	Sku [-]	Ra [µm]	Sq [µm]	Sp [µm]	Sv [µm]	Ssk [-]	Sku [-]
0.470 ± 0.052	0.554 ± 0.087	1.67 ± 0.01	1.71 ± 0.01	0.072 ± 0.094	2.97 ± 0.16	1.050 ± 0.059	1.32 ± 0.05	2.93 ± 0.03	2.95 ± 0.03	-0.108 ± 0.032	1.82 ± 0.08

For AP-X, the kurtosis parameter Sku was approximately 3 (Tables 3 and 4), indicating a relatively even distribution of surface peaks and valleys. In contrast, profile and axonometric images, together with negative skewness values (Ssk < 0), indicated a plateau-like surface topography for FSE.

The profile roughness parameter Ra and the surface roughness parameter Sq of FSE ranged from 0.965 to 1.050 μm and from 1.25 to 1.32 μm, respectively, whereas AP-X exhibited considerably lower values (Ra: 0.470–0.489 μm; Sq: 0.554–0.667 μm). Thus, the machined FSE surfaces were characterized by peaks and valleys nearly twice as high as those observed for AP-X.

**Table 5:** Machined surface morphology (SEM).

	AP-X	FSE
x2 000		
x50 000		

The SEM micrographs (Table 5 with detailed view) showed that AP-X presented a more isotropic surface structure with fewer irregularities, whereas FSE exhibited a rougher texture with irregular nanostructured peaks and valleys.

#### Two- and Three- Body Wear

Five specimens of each material were tested for each wear protocol (*n* = 5). Although the sample size was limited, the high level of standardization provided by the ACTA wear machine, where all specimens are mounted on a single rotating wheel and subjected to identical force, speed, and environmental conditions simultaneously, ensures high reproducibility and minimizes experimental variance. This setup allows for reliable statistical differentiation between materials even with a smaller sample size, as reflected in the low standard deviation values obtained.

Both AP-X and FSE exhibit similar mean wear rates under two-body wear conditions (full contact) (Table 6). A clear difference was observed under three-body wear conditions (lubricated contact with an abrasive medium), where the wear rate increased approximately 13-fold for AP-X and 8-fold for FSE compared with two-body wear protocol.

**Table 6:** Wear tests – mean wear rate WR.

Material	Two-Body wear WR [µm]	Three-Body wear WR [µm]
AP-X	1.9 ± 0.7	24.5 ± 1.1
FSE	1.9 ± 0.5	15.1 ± 0.7

The wear rates observed in this study for AP-X and FSE showed a high degree of consistency with previous research. Specifically, the three-body wear rates (24.5 μm for AP-X and 15.1 μm for FSE) closely mirror the results reported by Osiewicz et al. in 2019^15^, where the values for these materials were 23.4 μm and 14.5 μm, respectively. This remarkable reproducibility validates the stability of the ACTA wear simulator and confirms the established performance hierarchy between these two materials under abrasive conditions. These confirmed trends provide a solid baseline for the advanced 3D surface texture and SEM morphological evaluations conducted in the present work.

Quantitative analysis showed that while both materials exhibited identical wear rates in the two-body wear protocol (1.9 μm), their performance diverged significantly under three-body conditions. The nano-hybrid composite (FSE) demonstrated a 38.4% higher wear resistance than the micro-hybrid composite (AP-X). Specifically, the mean wear rate for FSE was 15.1 ± 0.7 μm, while for AP-X it was 24.5 ± 1.1 μm, indicating a clear advantage for the nano-cluster filler system in resisting three-body wear.

#### Worn surface texture

Following friction testing, both materials exhibited surface texture degradation, with patterns strongly dependent on the wear mode (two-body or three-body wear protocols). Qualitative 2D and 3D representations and quantitative surface texture parameters (Tables 7 and 8) confirmed these differences. Table 9 presents the SEM micrographs with descriptions of wear mechanisms.

**Table 7:** Surface texture characteristics – worn surface (after two-body wear protocol).

AP-X						FSE					
**3D view (surface topography)**											
	
**2D view (profile)**											
	
**Surface texture parameters**											
Ra [µm]	Sq [µm]	Sp [µm]	Sv [µm]	Ssk [-]	Sku [-]	Ra [µm]	Sq [µm]	Sp [µm]	Sv [µm]	Ssk [-]	Sku [-]
0.729 ± 0.038	0.861 ± 0.171	2.97 ± 0.05	1.86 ± 0.05	0.875 ± 0.434	4.21 ± 0.71	1.005 ± 0.005	1.59 ± 0.51	2.35 ± 0.05	4.80 ± 0.01	-1.580 ± 0.030	6.07 ± 0.01

**Table 8:** Surface texture characteristics – worn surface (after three-body wear protocol).

AP-X						FSE					
**3D view (surface topography)**											
	
**2D view (profile)**											
	
**Surface texture parameters**											
Ra [µm]	Sq [µm]	Sp [µm]	Sv [µm]	Ssk [-]	Sku [-]	Ra [µm]	Sq [µm]	Sp [µm]	Sv [µm]	Ssk [-]	Sku [-]
0.966 ± 0.153	0.869 ± 0.105	2.69 ± 0.01	2.70 ± 0.05	0.067 ± 0.027	3.27 ± 0.06	0.564 ± 0.039	0.766 ± 0.006	2.30 ± 0.02	2.70 ± 0.02	-0.301 ± 0.013	3.54 ± 0.07

**Table 9:**
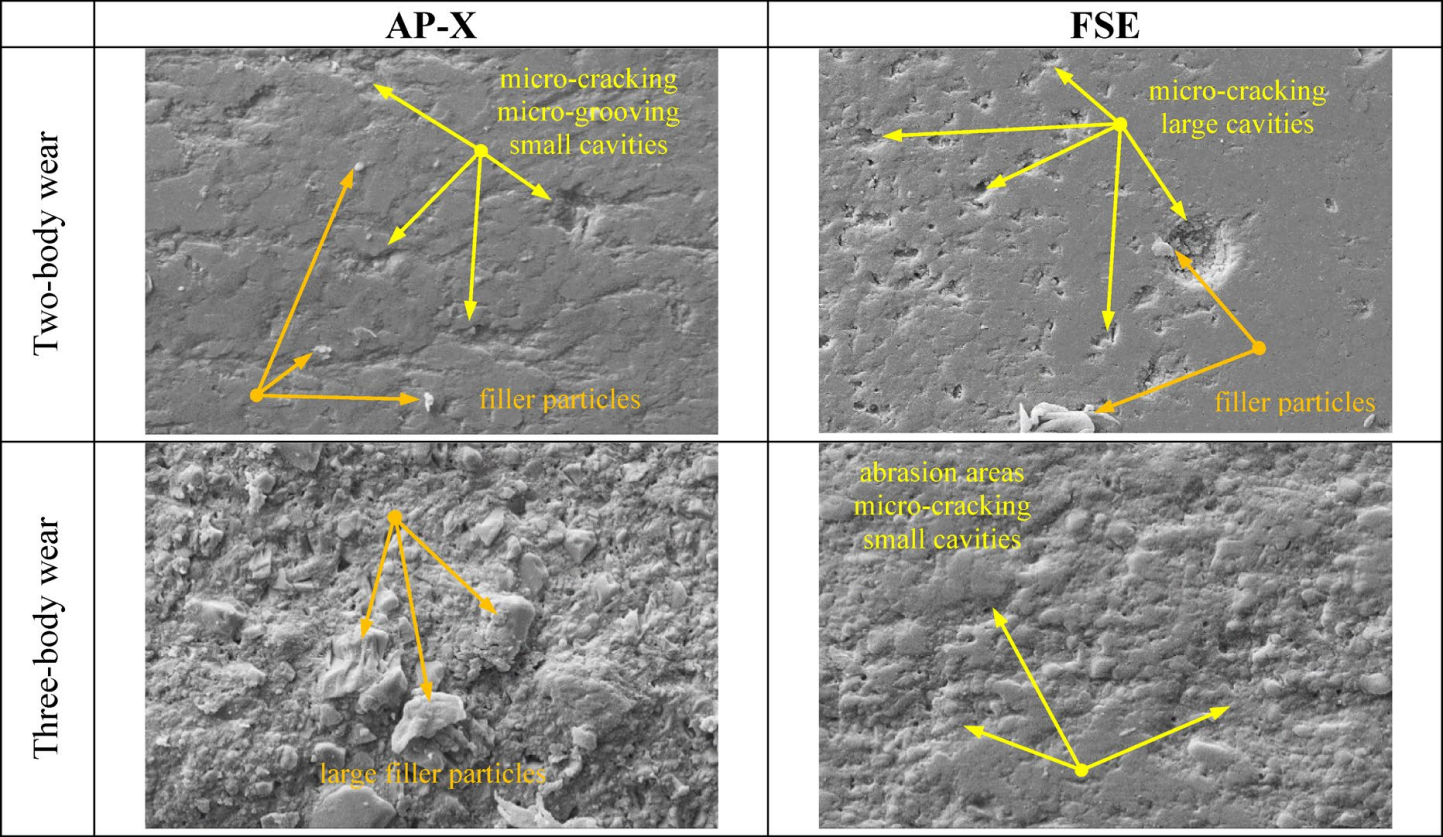
Worn surface morphology (SEM micrographs, magnification 2000x) after 200,000 cycles using the ACTA wear simulator protocols: two-body wear (demineralized water, 15 N contact load) and three-body wear (slurry of rice/millet seed shells in buffer, 15 N contact load).

#### Two-body wear

The surface topography analysis (Table 7) was consistent with SEM observations. Axonometric views and profiles revealed smaller cavities for AP-X and larger, deeper defects for FSE. For AP-X, mean value of the surface texture parameters increased markedly: Ra increased to 0.729 μm (1.5-fold) and Sq to 0.861 μm (1.3-fold). Other parameters (Sp, Ssk, and Sku) increased by at least 1.5-fold, whereas Sv remained approximately unchanged. For FSE, mean value of the surface texture parameters increased slightly: Ra increased to 1.005 μm (similar to the machined surface) and Sq to 1.59 μm (1.3-fold). Parameter Sp remained comparable to the machined surface, while Sv, Ssk, and Sku increased substantially. These findings indicate a deepening of the plateau-like surface character of FSE (Ssk < 0) and the formation of chaotically distributed wear traces and debris on the surface (Sku > 3).

The SEM observations of worn surfaces obtained under two-body wear (Table 9, same magnification) revealed clear wear tracks on both materials. AP-X surfaces were characterized by small cavities and fatigue micro-cracking/micro-grooving or abrasive scratching (indicated by yellow arrows) and exposed filler particles (indicated by orange arrows). FSE surfaces exhibited larger cavities, micro-cracking and more pronounced filler particle pull-out. These features were attributed to the characteristic of a lubricating medium under two-body contact.

#### Three-body wear

The surface topography analysis (Table 8) corresponded well with the SEM findings. Axonometric views and surface profiles demonstrated a more regular distribution of peaks and valleys on both materials, with a more pronounced plateau-like surface for FSE (Ssk < 0). For AP-X, mean value of the surface texture parameters increased: Ra to 0.966 μm (approximately two-fold) and Sq to 0.869 μm (1.6-fold), likely due to a displacement of larger filler particles under the abrasive action. Parameters Sp and Sv also increased (both approximately 1.6-fold), whereas Ssk and Sku remained similar to those of the machined surfaces.

In contrast, FSE exhibited a significant reduction in mean value of the surface texture parameters: Ra decreased to 0.564 μm (1.9-fold decrease) and Sq to 0.766 μm (1.7-fold decrease). Parameters Sp and Sv decreased slightly, whereas Ssk and Sku increased markedly (approximately three-fold and two-fold, respectively).

These results showed the transformation of the FSE surface toward a more pronounced plateau-like character (Ssk < 0) and the formation of an evenly distributed pattern of peaks and valleys associated with wear traces and wear debris (Sku > 3).

The SEM observations of worn surfaces obtained under three-body wear showed that the contrast is even more evident (Table 9, same magnification): AP-X displays a more worn but maintains structural integrity due to its high load of barium glass fillers. In contrast, FSE exhibited extensive ‘plucking’ of nanoclusters, resulting in a more irregular surface morphology. AP-X surfaces were characterized mainly by exposed large filler particles (indicated by orange arrows), whereas FSE surfaces showed predominantly small cavities and micro-cracking extensive abrasion areas (indicated by yellow arrows) without clearly showing the filler particles. This simultaneous comparison confirms that while both materials suffered more volume loss in three-body friction conditions, the nano-hybrid structure of FSE was more susceptible to filler loss than the micro-hybrid structure of AP-X.

The significantly higher volume loss observed in three-body wear conditions compared to two-body wear conditions can be attributed to the fundamental difference in wear mechanisms. In the two-body wear protocol, material degradation was primarily a result of direct contact-sliding fatigue, localized to the specific areas of occlusion. However, in the three-body protocol, the introduction of an abrasive slurry (millet and rice shells) introduced an erosive/abrasive component. These erosive/abrasive particles acted as independent intermediaries that transfer the load across a broader surface area, effectively ‘scouring’ the resin matrix^16^. That process facilitated the ‘plucking’ of filler particles and led to generalized surface loss. As established by De Gee and Pallav^12^, the three-body medium replicates the abrasive nature of food during mastication, which consistently produces higher wear rates than the cleaner, water-lubricated conditions of two-body contact.

## Discussion

The presented studies combines quantitative surface topography analysis and SEM-based morphology evaluation. Before the wear tests, AP-X exhibited smoother machined surfaces (Ra = 0.470–0.489 μm) compared to FSE (Ra = 0.965–1.050 μm), likely due to its high filler load and larger particle morphology. The SEM micrographs showed that AP-X had a relatively uniform and dense filler distribution, whereas the FSE displayed a more heterogeneous nanocluster-based surface morphology.

The friction test results clearly demonstrated that the presence of an abrasive medium dramatically increased material loss: the mean wear rate WR increased from approximately 1.9 μm in the two-body friction conditions to 24.5 μm for AP-X and 15.1 μm for the FSE in the three-body friction conditions, representing approximately 13- and 8-fold increases in wear, respectively.

Under the full contact friction conditions (two-body wear), mechanisms related to direct friction and cyclic shearing of surface-layer asperities dominated. Micro-grooving and local degradation of the matrix and filler–matrix interfaces occurred.

FSE exhibited surface degradation characterized by increased roughness (Ra = 1.005 μm, Sq = 1.59 μm), highly negative skewness (Ssk = − 1.580), and elevated kurtosis (Sku = 6.07). SEM micrographs confirmed the presence of matrix micro-cracking and nanocluster displacement, consistent with findings by Maier et al.^17^. Those features were indicative of fatigue-driven deterioration and possible interfacial failure between filler clusters and the resin, potentially exacerbated by internal stresses from polymerization or incomplete silanization^18^.

In contrast, AP-X displayed a less severe response to the two-body friction conditions in terms of increases in roughness (Ra = 0.729 μm and Sq = 0.861 μm) and showed less evidence of micro-cracking or filler–matrix separation in the SEM micrographs. This resilience may be attributed to its micro-hybrid architecture, which distributed stress more uniformly.

The significantly higher wear observed under the slurry-lubricated friction conditions (three-body wear) resulted from a qualitative change in the nature of contact: suspension particles, repeatedly drawn into the contact zone, transferred part of the load and acted as moving micro-tools, causing intense micro-cutting/micro-shearing^19,20^ of the matrix and undercutting of the matrix around the filler, which facilitated its breakout and the generation of defects.

Under the slurry-lubricated friction conditions (three-body wear), FSE showed a polishing effect, evidenced by reduced roughness (Ra = 0.564 μm, Sq = 0.766 μm), likely due to fine filler interaction with the abrasive medium. Despite the persistently high WR, the material removal was more uniform and led to smoothing of the surface unevenness rather than reducing volumetric loss. The SEM micrographs showed smoother worn surfaces with limited micro-cracking.

Conversely, the AP-X displayed increased roughness (Ra = 0.966 μm, Sq = 0.869 μm), suggesting that its larger fillers were more susceptible to removal by slurry-based abrasion, generating craters and secondary roughness increases, which may accelerate further abrasion.

A material-specific filler composition likely contributed to these differences. FSE contained zirconia–silica nanoclusters, whereas AP-X incorporated barium glass particles. Zirconia was significantly harder than barium glass. While higher hardness increases abrasion resistance, it may also lead to localized stress accumulation and micro-cracking formation when bonding is insufficient. Barium glass, being softer and more uniformly distributed, may better absorb and distribute mechanical loads, reducing crack initiation. As Ferracane^1^ emphasized, bulk properties such as surface hardness are not reliable predictors of wear resistance. Instead, long-term durability is influenced by the synergy between filler morphology, resin bonding, and micromechanical behaviour—factors best assessed using tools such as SEM.

The distinction between the two-body and three-body friction conditions simulations holds important implications for patient-specific material selection. Bruxism, characterized by repetitive direct tooth contact, mirrors the two-body wear conditions and leads to fatigue-driven degradation. In bruxing patients, restorations may appear more polished due to surface flattening but may be internally weakened by subsurface micro-cracking^21,22^. In contrast, the non-bruxers typically experience three-body wear, involving saliva and food as third-body abrasives. This results in more uniform surface abrasion and roughening over time, which may affect aesthetics but is less likely to induce subsurface damage^12^. These functional distinctions may explain differences in clinical outcomes between bruxers and non-bruxers and help guide clinicians in the material selection. The AP-X, with its fatigue resistance and stable surface morphology, may be better suited for the bruxers. FSE, with its polish retention and smoother wear profile under the lubricated conditions, may be more appropriate for patients without parafunctional habits.

## Conclusions

The conducted research and analysis of the results allowed the formulation of the following conclusions:

- Surface texture analysis is important for characterizing machined and worn surfaces, including their features (qualitative analysis) and roughness parameters (quantitative analysis)^23,24^.
- The mean wear rate (WR) was greater under the three-body friction conditions than under two-body friction conditions, which was reflected in worn surface texture and wear mechanisms.
- FSE (nano-hybrid) exhibited greater surface degradation under the two-body friction conditions due to nanocluster displacement (large cavities and matrix micro-cracking), whereas under the three-body friction conditions FSE showed less surface degradation (extensive abrasion areas, small cavities and micro-cracking).
- AP-X (micro-hybrid) showed better resistance to fatigue-related micro-cracking/micro-grooving or abrasive scratching under the two-body friction conditions but experienced increased surface roughness under the three-body friction conditions.
- Zirconia-based fillers (in FSE) are harder and more wear-resistant but may promote interfacial stress and cracking, whereas barium glass fillers (in AP-X) distribute stress more evenly, enhancing fatigue resistance.
- Clinical implications suggest that AP-X may be more suitable for bruxers, while FSE may offer better performance in patients with standard masticatory function.

## Data Availability

The datasets used and analysed during the current study available from the corresponding author on reasonable request.
